# *APOE* alleles’ association with cognitive function differs across Hispanic/Latino groups and genetic ancestry in the study of Latinos-investigation of neurocognitive aging (HCHS/SOL)

**DOI:** 10.1002/alz.12205

**Published:** 2020-11-06

**Authors:** Einat Granot-Hershkovitz, Wassim Tarraf, Nuzulul Kurniansyah, Martha Daviglus, Carmen R. Isasi, Robert Kaplan, Melissa Lamar, Krista M. Perreira, Sylvia Wassertheil-Smoller, Ariana Stickel, Bharat Thyagarajan, Donglin Zeng, Myriam Fornage, Charles S. DeCarli, Hector M. González, Tamar Sofer

**Affiliations:** 1 Division of Sleep and Circadian Disorders, Department of Medicine, Brigham and Women’s Hospital, Boston, Massachusetts, USA; 2 Department of Medicine, Harvard Medical School, Boston, Massachusetts, USA; 3 Institute of Gerontology, Wayne State University, Detroit, Michigan, USA; 4 Department of Medicine, Institute for Minority Health Research, University of Illinois at Chicago, Chicago, Illinois, USA; 5 Department of Epidemiology & Population Health, Department of Pediatrics, Albert Einstein College of Medicine, Bronx, New York, USA; 6 Division of Public Health Sciences, Fred Hutchinson Cancer Research Center, Seattle, Washington, USA; 7 Rush Alzheimer’s Disease Research Center, Rush University Medical Center, Chicago, Illinois, USA; 8 Department of Social Medicine, University of North Carolina School of Medicine, Chapel Hill, North Carolina, USA; 9 Department of Neurosciences and Shiley-Marcos Alzheimer’s Disease Center, University of California, San Diego, La Jolla, California, USA; 10 Department of Laboratory Medicine and Pathology, University of Minnesota, Minneapolis, Minnesota, USA; 11 Department of Biostatistics, Gillings School of Global Public Health, University of North Carolina, Chapel Hill, North Carolina, USA; 12 Institute of Molecular Medicine, The University of Texas Health Science Center at Houston, Houston, Texas, USA; 13 Department of Neurology, Center for Neuroscience, University of California at Davis, Sacramento, California, USA; 14 Department of Biostatistics, Harvard T.H. Chan School of Public Health, Boston, Massachusetts, USA

**Keywords:** admixture, Alzheimer’s disease, ancestry, apolipoprotein E, cognitive decline, genetic epidemiology, Hispanics/Latinos, mild cognitive impairment

## Abstract

**Introduction:**

Apolipoprotein E (*APOE*) alleles are associated with cognitive decline, mild cognitive impairment (MCI), and Alzheimer’s disease in Whites, but have weaker and inconsistent effects reported in Latinos. We hypothesized that this heterogeneity is due to ancestry-specific genetic effects.

**Methods:**

We investigated the associations of the *APOE* alleles with significant cognitive decline and MCI in 4183 Latinos, stratified by six Latino backgrounds, and explored whether the proportion of continental genetic ancestry (European, African, and Amerindian) modifies these associations.

**Results:**

*APOE ε*4 was associated with an increased risk of significant cognitive decline (odds ratio [OR] = 1.15, *P*-value = 0.03), with the strongest association in Cubans (OR = 1.46, *P*-value = 0.007). *APOE-ε*2 was associated with decreased risk of MCI (OR = 0.37, *P*-value = 0.04) in Puerto Ricans. Amerindian genetic ancestry was found to protect from the risk conferred by *APOE ε*4 on significant cognitive decline.

**Discussion:**

Results suggest that *APOE* alleles’ effects on cognitive outcomes differ across six Latino backgrounds and are modified by continental genetic ancestry.

## 1 | BACKGROUND

Cognitive decline, mild cognitive impairment (MCI), and Alzheimer’s disease and related dementias (ADRD) are a growing worldwide epidemic and one of the leading causes of death in the elderly population.^1^ MCI is a prodromal cognitive impairment state preceding the more serious cognitive dysfunction characteristic of dementia. It can involve problems with memory, language, thinking, and judgment that are greater than normal age-related changes.^2,3^ Cognitive decline is a normal process of aging; however, in ADRD patients it begins many years before dementia is diagnosed and accelerates during the course of the disease.^3^ Self-reported cognitive decline has lately been introduced to the field, to extend ADRD risk diagnosis to an earlier stage before MCI.^4^ Hispanics/Latinos (Latinos henceforth) are the fastest-growing ethnic group in the United States,^5^ and suffer from higher rates of ADRD, compared to Whites.^6,7^

The apoplipoprotein E (*APOE*) *ε*4 allele is the strongest known genetic risk factor for ADRD,^8^ and it has also been linked to more rapid cognitive aging, such as increased cognitive decline and MCI.^9–11^ The *APOE* gene exists as three polymorphic alleles*ε*2, *ε*3, and *ε*4which are determined by two single nucleotide polymorphisms (SNPs: rs429358 and rs7412), that substitute amino acids in the protein, resulting in functional changes. In general, *APOE ε*4 confers increased risk for cognitive decline, MCI, and ADRD compared to the more common *APOE ε*3, whereas the *APOE ε*2 is considered neuroprotective.^12^ However, most of these findings are based on studies of individuals of European ancestry. Several population-based studies have shown ancestry heterogeneity of the *APOE ε*4ADRD association.^13–15^ By comparing haplotype *ε*4/*ε*4 to haplotype *ε*3/*ε*3, Farrer et al.^8^ reported strong effects in Japanese (odds ratio [OR] = 33.1), and Whites (OR = 12.5), and weaker effects among African Americans (OR = 5.7) and Latinos (OR = 2.2). Other studies investigating the association of *APOE ε*4 with MCI and dementia in Latinos have produced inconsistent results.^16–20^ A study of Mexican Americans indicated that haplotype *ε*4/*ε*4, compared to haplotype *ε*3/*ε*3, was associated with lower cognitive scores and higher dementia, though not significant (risk ratio [RR] = 2.04, confidence interval [CI] = 0.88–4.72).^16^ Similarly, two other studies suggested that the *APOE ε*4 allele is both less common and confers less risk for MCI or ADRD in Mexican Latinos compared to Whites.^17,18^ However, another study in Caribbean Latinos (ie, Dominicans and Puerto Ricans) showed that late-onset familial Alzheimer’s disease (AD) is strongly associated with *APOE ε*4, with the *APOE ε*4 allele more likely to be transmitted among affected individuals than unaffected relatives.^19^ These inconsistent results may be due, in part, to small sample sizes, different study designs and samples, and definitions of cognitive outcome,^20^ as well as to the heterogeneity of the Latino groups in which the studies were conducted.

The Hispanic Community Health Study/Study of Latinos (HCHS/SOL) is a population-based longitudinal cohort study of 16,415 U.S. Hispanic/Latino adults that enrolled participants from Cuban, Central American, Dominican, Mexican, Puerto Rican, and South American backgrounds.^21,22^ Previous characterization of the genetic diversity in the HCHS/SOL cohort has shown that Latino individuals have admixed genomes consisting of three predominant continental ancestries: Amerindian, European, and African with varying proportions among and within each background group.^23^ Furthermore, it was shown that the *APOE* alleles have different distributions among the six Latino background groups,^20^ consistent with different *APOE* allele frequencies among the ancestral populations.

Previous association analysis of the *APOE* alleles with MCI in the HCHS/SOL ancillary Study of Latinos-Investigation of Neurocognitive Aging (SOL-INCA) did not detect significant associations.^24^ We hypothesized that there are differential association effects of the *APOE* alleles in the six Latino background groups on significant cognitive decline and MCI, which could potentially explain the inconsistent reported literature results of the association of *APOE* alleles with cognitive function, MCI, and dementia in Latinos. To address this, we tested the association of *APOE* alleles with significant cognitive decline and MCI and stratified the analyses by the six Latino background groups. Next, we hypothesized that differences in proportions of continental ancestries in the six Latino background groups will explain the heterogeneous *APOE* alleles’ effects on significant cognitive decline and MCI. The purpose of this study was to determine whether genetic ancestry modifies the effect of *APOE* alleles on the significant cognitive decline and MCI by testing interaction effects between the three Latino continental ancestries and the *APOE* alleles.

## 2 | METHODS

### 2.1 | Study population

The HCHS/SOL is a multisite, prospective cohort study of diverse Latinos enrolled at four field centers (Bronx, New York; Chicago, Illinois; Miami, Florida; and San Diego, California). The sampling design details have been previously described.^21,22^ A total of 16,415 self-identified Hispanic/Latino adults, 18 to 74 years old, were enrolled at HCHS/SOL baseline visit 1 (2008–2011). Anthropometry, biospecimens, and health information about risk/protective factors were collected. The baseline cognitive battery included four tests: Six-Item Screener (SIS; mental status);^25^ Brief-Spanish English Verbal Learning Test (B-SEVLT; verbal episodic learning and memory);^26^ Word Fluency;^27^ and Digit Symbol Substitution test (DSS; processing speed, executive function). HCHS/SOL visit 2 occurred between 2014 and 2017 with an abbreviated protocol. SOL-INCA is an HCHS/SOL ancillary study that occurred at visit 2, and included the oversampled middle-aged and older participants (50 years and older) who were administered the visit 1 cognitive battery, plus additional complementing cognitive tests to determine self-reported measures of cognitive decline (Everyday Cognition-12 [E-Cog12])^28^ and functional status (Instrumental Activities of Daily Living Scale [IADL]), among other cognitive tests.^29^ Overall, 6377 participants were re-examined in SOL-INCA after an average of 7 years since their visit 1 cognitive assessment. Of the 6377 participants, 2127 were excluded from analyses (1688 did not consent for genetic data, 420 failed *APOE* genotyping, 67 had missing cognitive outcomes, and 19 had missing covariates), totaling an analytic sample of 4183 individuals (mean age = 62.1 years and 52.5% were women). All participants in this analysis signed informed consent in their preferred language (Spanish/English) to use their genetic and non-genetic data. The study was reviewed and approved by the institutional review boards at all collaborating institutions.

### 2.2 | Cognitive outcomes

We analyzed two binary cognitive variables that were previously constructed^30^ based on cognitive tests and self-report: significant cognitive decline, and MCI. Cognitive decline measures the cognitive decline between HCHS/SOL visit 1 and the SOL-INCA exam, based on a latent factor model taking into account cognitive test scores. Individuals were classified as meeting significant cognitive decline criteria if they had a change in global cognitive performance between the two exams that exceeded –0.055 standard deviation (SD) yearly.^30^

Individuals were classified as MCI according to National Institute on Aging-Alzheimer’s Association if they fit the following three criteria:^31^ (1) a cognitive test score below −1SD based on SOL-INCA robust internal norms, (2) significant cognitive decline (described above), and (3) self-reported cognitive decline based on the E-Cog12.^28^ Also, individuals that had both a cognitive deficit < −2SD in any neurocognitive test at the SOL-INCA exam and more than minimal cognitive impairment in the IADL scale, were classified as MCI. Additional information about the SOL-INCA cognitive assessment approach is provided in detail in Appendix 1 of a previous publication.^30^

### 2.3 | Genetic data

Genotyping, quality control, and continental ancestry inference were previously described.^23,32^ In brief, genotyping was performed using Illumina custom array and genome-wide imputation was conducted with the 1000 Genome Project reference panel.^32^ Principal components (PCs) were estimated using PC-Relate^33^ and continental-ancestry proportions were calculated using ADMIXTURE software.^23,34^ “Genetic analysis groups” were constructed based on a combination of self-identified Hispanic/Latino background and genetic similarity, and are classified as Cuban, Dominican, and Puerto Rican (Caribbean groups); and Mexican, Central American, and South American (Mainland groups).^23^ *APOE* genotyping was performed using commercial TaqMan assays previously described.^20^ *APOE* variants were in Hardy-Weinberg equilibrium (*P* > 0.05).

### 2.4 | Statistical analysis

We provided descriptive statistics to characterize the demographic and cognitive outcomes and *APOE* allele distributions in the full analytic dataset and by background groups. We tested the associations between *APOE ε*2 and *ε*4 alleles with cognitive outcomes in the same model (*APOE ε*3 used as the reference allele) using a complex survey design from the R “survey” package,^35^ with a “quasipoisson” family for binary traits. This method accounts for the stratification, clustering, and probability weighting in HCHS/SOL to allow correct generalizations to the target population of U.S. Latinos. Models were adjusted for age, sex, education, center, first five genetic PCs, and “genetic analysis groups.” We tested both an additive and a dominant inheritance mode of the *APOE* alleles. For a given *APOE* allele, additive inheritance mode counts the number of the alleles for each individual (0, 1, or 2); dominant mode 1 if an individual had at least one allele, 0 otherwise. Carriers of both alleles ie, *APOE ε*2/*ε*4 were included in the model by giving a value 1 for the *APOE ε*2 variable and a value of 1 for the *APOE ε*4 variable (for both modes of inheritance).

Further association analyses of *APOE ε*4 and *ε*2 alleles with cognitive outcomes were done separately for each of the six genetic analysis groups and the significance of the results was evaluated through 10,000 permutations for each group, to protect from potential high type 1 error due to the low proportion of *APOE* variants. Effect modification by genetic analysis groups was tested by including multiplicative interaction terms of these groups with the *APOE* alleles followed by a Cochran’s Q heterogeneity test accounting for correlations between effect estimates. Continental-ancestry proportion interaction with *APOE* alleles effects was tested separately for each of the three ancestries (European, African, and Amerindian), by including the continental-ancestry proportion variable together with a multiplicative interaction term of ancestry with the *APOE* allele (analytic dataset n = 3618).^23^

Power calculation analysis for the two cognitive outcomes (cognitive decline and MCI) was calculated using population risk allele frequencies.^36^ For *APOE ε*4 association with cognitive decline, the effect size was estimated based on the Cuban group and for *APOE ε*4 association with MCI, the effect size was estimated based on the Puerto Rican group, because these groups showed significant effects. This calculation did not account for age distribution in the background groups.

## 3 | RESULTS

Table 1 presents summary statistics of the SOL-INCA analytic sample used in this article, including sample size, distribution of sex, age, education, measures of cognitive function, distribution of the *APOE* alleles, and genetic proportion ancestries by genetic background groups. Overall, our dataset included 4183 participants (1568 men; 2615 women), with a mean age of 62.1 years. Figure S1 in supporting information illustrates the overlap between the two dichotomous cognitive outcomes. Thirty percent of the population was classified with significant cognitive decline (n = 1250); of these, one third were also classified as MCI (n = 430). Twelve more participants have solely MCI.

The distribution of the *APOE* alleles in our SOL-INCA analytic sample by genetic background groups demonstrates the differential distribution, similar to the results published by González et al.^20^ Overall, *APOE ε*3 is the most frequent allele in all Latino background groups, while *ε*4 and *ε*2 are relatively rare. Table 2 summarizes the associations between the *APOE* alleles and cognitive outcomes in the SOL-INCA population based on an additive inheritance model. The OR for cognitive decline with one *APOE ε*4 allele, compared to individuals without *APOE ε*4 allele, was 1.15 (95% CI [1.01;1.32], *P*-value = 0.03); restriction to older subsets of the SOL-INCA population resulted in stronger association (ORs = 1.15–1.31; Table S1 in supporting information). However, no significant associations were obtained between *APOE ε*4 allele and MCI, and between *APOE ε*2 allele and both cognitive outcomes in the total SOL-INCA analytic sample.

We further stratified and explored the association of the *APOE* alleles with cognitive outcomes in the six background groups: Cuban, Dominican, Mexican, Puerto-Rican, South-American, and Central-American, summarized in Table 3 (additive model). The association between the *APOE ε*4 allele with cognitive decline remained significant only for Cubans showing a stronger effect compared to the effect in the total population (OR = 1.46, 95% CI [1.13;1.88], *P*-value = 0.007). Additionally, stratification to Latino background groups also revealed a new nominally significant protective association between the *APOE ε*2 allele and MCI in the Puerto Rican group (OR = 0.37, 95% CI [0.16;0.84], *P*-value = 0.04). Heterogeneity tests for differences between the background group association tests were not significant.

Tables S2 and S3 in supporting information present similar associations between *APOE* alleles and cognitive outcomes in the SOL-INCA analytic sample, based on a dominant inheritance mode. Power analysis for the cognitive traits is presented in Table S4 in supporting information. Power for the *APOE* allele associations with cognitive functions is substantially different among the six Latino backgrounds (ranges: MCI 0.34–1.00, cognitive decline 0.26–0.88).

**FIGURE 1:**
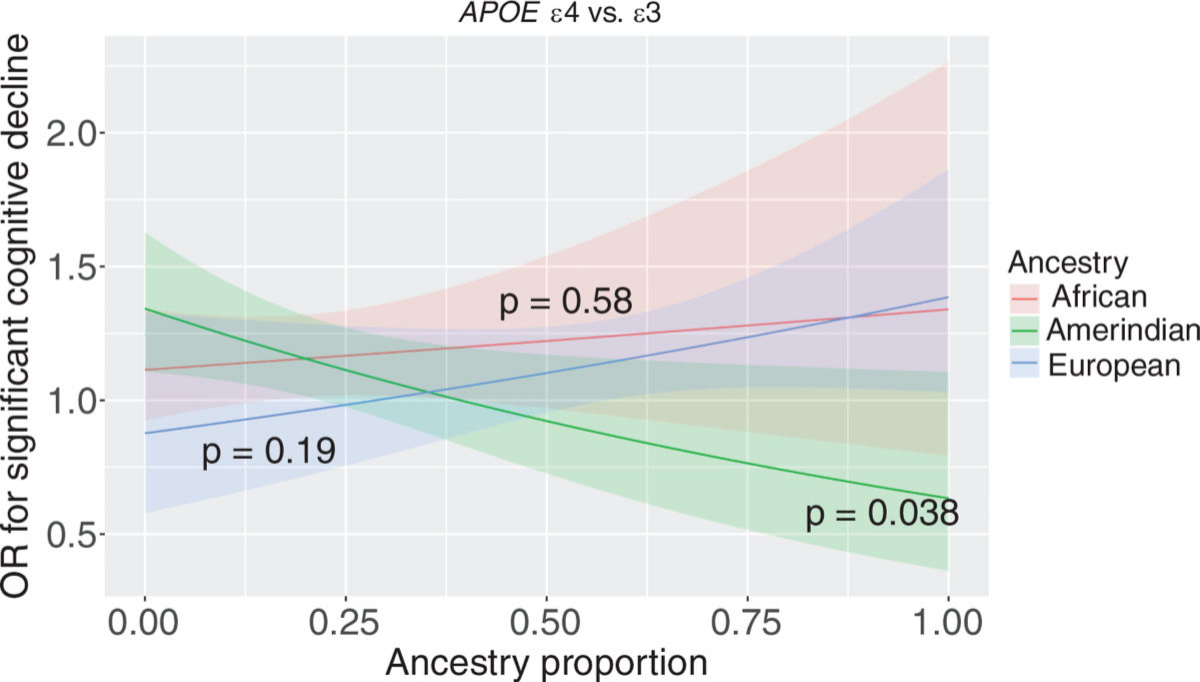
Interaction of proportion continental ancestry with apolipoprotein E(*APOE*) *ε*4 allele effects on significant cognitive decline. Interaction *P*-values were estimated based on 10,000 permutations for each ancestry separately

Proportion ancestry interaction with *APOE* alleles’ effects on cognitive outcomes is presented in Table 4. A significant interaction effect was found between Amerindian ancestry and *APOE ε*4 on cognitive decline (OR = 0.47, 95% CI [0.24;0.93], *P*-value = 0.04), such that protection from the risk of cognitive decline in *APOE ε*4 carriers was associated with higher Amerindian proportion ancestry (Figure 1).

Additional analyses of continuous cognitive decline are reported in Appendix 1 and show similar results to the significant cognitive decline outcome.

## 4 | DISCUSSION

We performed an association study of *APOE* alleles and cognitive outcomes in a large cohort of diverse middle-aged and older Latinos. Our main result showed an association between the *APOE ε*4 allele and the risk of significant cognitive decline. In the stratified analysis, this result remained significant only for Cubans. We also found an association between *APOE ε*2 and decreased risk of MCI only in Puerto Ricans, suggesting differential effects of the *APOE* alleles on cognitive function in the Latino background groups. We further discovered that an increased proportion of genetic Amerindian ancestry was associated with a protective effect from the risk of *APOE ε*4 on significant cognitive decline, compatible with the known low proportion of Amerindian ancestry in the Cuban background group.^23^

We infer that ancestry-specific genetic variants may explain the differential effects of *APOE* alleles in the six Latino backgrounds.

The association of *APOE ε*4 allele with significant cognitive decline (OR = 1.15) is compatible with previous results presenting a relatively weaker association between *APOE ε*4 allele and ADRD in the Latino population compared to Whites (OR = 2.2).^8^ The difference in effect sizes may be due to the difference in the tested phenotypes, significant cognitive decline versus ADRD. Cognitive decline modeling differs substantially between studies, thus a comparison of effect sizes of *APOE* alleles on significant cognitive decline is not feasible.^37^ In the analysis stratified by “genetic analysis groups,” we anticipated some relationship between *APOE ε*4 and significant cognitive decline specifically among Cubans because they have higher degrees of European ancestry.^23^ Cubans in our analytic dataset are also older (a risk factor for cognitive decline) and more educated (a protective factor for cognitive decline) compared to the other Latino backgrounds; however, our models controlled for age and education. A previous study conducted in Cubans from Cuba also reported an association between *APOE ε*4 and incident of dementia with a stronger effect in middle-aged adults (<70 years) compared to older adults (>70 years).^38^

The non-significant results for the *APOE ε*4 allele association with significant cognitive decline in the five other Latino backgrounds are consistent with their lower percentage of European ancestry.^23^ This could also result from the predominantly middle-aged SOL-INCA population (mean age 62.1 years), a population not fully presenting with significant cognitive decline, whereas most epidemiological studies on *APOE* and cognitive decline outcomes are conducted in individuals ≥65 years. This claim is supported by analysis restricted to older subsets of the population resulting in a stronger association between *APOE ε*4 allele and significant cognitive decline in the older subsets (Table S1). Alternatively, it may result from limited statistical power eg, power = 0.26 for the South American group for the cognitive decline trait (Table S4), or it could present a true differential effect size of *APOE ε*4 on significant cognitive decline in the different Latino backgrounds. The latter hypothesis is supported by the fact that the statistical power for the Mexican group for the association of *APOE ε*4 with cognitive decline status is 0.88, yet not significant. Three previous studies focusing on Mexican-origin Latinos also showed the non-significant result with a relatively weak effect of *APOE ε*4 allele on dementia, MCI, or AD.^16–18^

Unique to this study, continental-ancestry proportion, which captures genetic variation across the genomes, further revealed that an increased proportion of genetic Amerindian ancestry was associated with a protective effect from the risk of *APOE ε*4 on significant cognitive decline. This result is also compatible with the *APOE ε*4 significant risk effect we found in Latino Cubans, which were shown to have the lowest proportion of Amerindian ancestry among all Latino background groups (Table 1). By using all three ancestries of the admixed Latino population we could infer the protective effect of the Amerindian ancestry, rather than the European or African ancestries being harmful (if there were only two studied ancestries, we could not distinguish between the protective effect of one and the risk effect of the other). This result is inconsistent with a recent report in Peruvians (78 AD cases and 128 controls) suggesting that Amerindian local ancestry in the *APOE* region is contributing to a strong risk for AD in *APOE ε*4 carriers.^39^ Two other studies in Caribbean Hispanics suggest a protective effect of the African local ancestry in the *APOE* region from the risk of *APO ε*4 on AD.^40,41^ Therefore, we also studied whether local ancestry at the *APOE* region modifies the effects of *APOE* on significant cognitive decline (results not shown). While the Amerindian local ancestry association was protective, it was not statistically significant (*P*-value >0.2). It is a topic of future research to perform a more comprehensive analysis of local ancestry at an expanded region around the *APOE* gene and potentially genome-wide, and search for specific genetic variants explaining the observed interaction of global Amerindian genetic ancestry with *APOE ε*4 in its effect on significant cognitive decline.

Our study also highlights a protective association between *APOE ε*2 and MCI solely in the Puerto Rican background group (Table 3), compatible with the known neuroprotective effect of *APOE ε*2.^8^ The direction of this effect was similarly protective in several other Latino background groups; however, they were not significant, despite high power estimations especially for the Cuban and Mexican background groups (Table S4). *APOE ε*2 is relatively rare and its effect on cognitive function is less studied compared to *APOE ε*4,^42^ specifically in the Latino population. A meta-analysis based predominantly on the Chinese population suggests that *APOE ε*2/*ε*3 genotype provides slight protection for MCI.^43^

We did not observe the expected risk effects of the *APOE ε*4 alleles with MCI. This could be explained by our middle-aged SOL-INCA population, not fully presenting MCI. While both significant cognitive decline and MCI are markers for ADRD, our results might indicate that significant cognitive decline, which appears in an earlier stage before MCI, is an important risk marker for ADRD in this SOL-INCA dataset.^44^

This is the first study to examine Latino genetic diversity in the context of significant cognitive decline and MCI that used both background groups and continental genetic ancestry proportions, which is a major step forward in the field of cognitive aging and ADRD precision medicine. Second, this cohort is composed of Latino middle-aged and older adults, thus there is less significant survival bias compared to studies of older adults. However, this dataset does not include biomarkers such as advanced imaging or fluid biomarkers, which could have validated the significant cognitive decline and MCI status. Also, we note that significant cognitive decline measure is not a clinical phenotype. To address this limitation, we report a similar analysis of *APOE* alleles with continuous cognitive decline (Appendix 1), and the results are similar to the results of significant cognitive decline. Because we examined six different Latino background groups, Winner’s curse bias might also explain our significant result in the stratified analyses. Overall, our differential association results in the Latino background groups may suggest a true differential genetic association between *APOE* alleles and cognitive outcomes related to admixed genomes. However, they could alternatively represent the lifestyle and environmental factors differing between the Latino ancestries, such as smoking, nutrition, alcohol consumption, physical activity, sleep phenotypes, air pollution, and metal exposure, that may also interact with *APOE* alleles and cognitive function risk.^45^ Further analysis in SOL-INCA and other samples with older Latino populations, accounting for environmental characteristics, may further delineate the associations between the *APOE* alleles and cognitive outcomes.

Overall, our study, together with other studies focusing on the Latino population, may lead to a better understanding of the role of *APOE* in the development of ADRD in Latinos and potentially in American Indians by extension thus advancing the development of personalized risk prediction and strategies to address Latinos’ health disparities in neurodegenerative aging and disorders.

## Supplementary Material

Supplementary_Table_1

Appendix_1

Supplementary_Figure_1

Supplementary_Table_2

Supplementary_Table_3

Supplementary_Table_4

## Figures and Tables

**TABLE 1:** Demographics, neurocognitive, and genetic characteristics of SOL-INCA by genetic background groups

		Cuban	Dominican	Mexican	Puerto Rican	South American	Central American	Overall
**N**		875	424	1411	734	313	417	4183
**Sex (%)**	**Female**	490 (48.1)	300 (60.5)	902 (53.1)	447 (50.9)	192 (56)	277 (58.4)	2,615 (52.5)
**Age in years**	**Mean (SD)**	62.6 (8.58)	61.65 (8.19)	61.7 (7.57)	62.9 (8.12)	61.96 (8.13)	61.35 (7.39)	62.08 (8.18)
**Age (%)**								
	**50–59**	342 (31.6)	192 (40.9)	642 (45.7)	281 (35.2)	139 (39.3)	182 (42.4)	1782 (38.4)
	**60–69**	364 (32.3)	162 (37.3)	547 (35.5)	306 (35.6)	123 (35.7)	184 (39.6)	1690 (35)
	**70+**	169 (36.1)	70 (21.8)	222 (18.8)	147 (29.2)	51 (25.1)	51 (18)	711 (26.6)
**Education (%)**								
	**< 12**	208 (24)	191 (43)	686 (46.1)	305 (42.1)	65 (18.3)	162 (39.5)	1622 (36)
	**12**	226 (24.5)	84 (20.1)	283 (21.4)	164 (20.7)	64 (18.2)	83 (17.8)	906 (21.7)
	**> 12**	441 (51.6)	149 (36.9)	442 (32.5)	265 (37.3)	184 (63.5)	172 (42.7)	1655 (42.3)
**Neurocognitive traits (%)**								
	**Significant cognitive decline**	232 (27.9)	140 (32)	436 (32.5)	241 (37.4)	87 (27.4)	113 (28.6)	1250 (31.2)
	**MCI**	85 (11.1)	47 (11.8)	148 (10.6)	87 (13.4)	28 (8.3)	46 (12.3)	442 (11.3)
***APOE* genotype (%)**								
	***ε*2/*ε*2**	2 (0.1)	4 (1)	4 (0.2)	2 (0.3)	0 (0)	1 (0)	14 (0.3)
	***ε*2/*ε*3**	96 (10)	56 (13.8)	70 (4.1)	60 (8.2)	20 (4.9)	27 (6.5)	330 (7.8)
	***ε*2/*ε*4**	14 (1.2)	14 (4)	7 (0.8)	11 (1.3)	2 (0.7)	3 (0.9)	51 (1.3)
	***ε*3/*ε*3**	570 (66.3)	231 (56)	1059 (76.2)	490 (68.1)	235 (78.4)	303 (72.3)	2893 (69.5)
	***ε*3/*ε*4**	178 (20.6)	107 (22.6)	253 (17.2)	163 (21.3)	46 (13.4)	75 (18.1)	824 (19.4)
	***ε*4/*ε*4**	15 (1.7)	12 (2.5)	18 (1.5)	8 (0.9)	10 (2.6)	8 (2.2)	71 (1.7)
***APOE* allele (%)**								
	***ε*2**	114 (0.06)	78 (0.10)	85 (0.03)	75 (0.05)	22 (0.03)	32 (0.04)	409 (0.05)
	***ε*3**	1414 (0.82)	625 (0.74)	2441 (0.87)	1203 (0.83)	536 (0.88)	708 (0.85)	6940 (0.83)
	***ε*4**	222 (0.13)	145 (0.16)	296 (0.11)	190 (0.12)	68 (0.10)	94 (0.12)	1017 (0.12)
**Proportion genetic ancestry (mean [SD])**								
	**N**	780	358	1194	641	272	364	3618
	**African**	0.16 (0.20)	0.46 (0.16)	0.04 (0.02)	0.22 (0.13)	0.07 (0.08)	0.10 (0.06)	0.16 (0.18)
	**European**	0.80 (0.21)	0.48 (0.15)	0.46 (0.20)	0.65 (0.12)	0.50 (0.22)	0.46 (0.15)	0.60 (0.24)
	**Amerindian**	0.05 (0.04)	0.06 (0.02)	0.49 (0.20)	0.13 (0.03)	0.43 (0.23)	0.44 (0.15)	0.24 (0.24)

Note: (%) based on the sampling weights and complex survey design.

Abbreviations: APOE, apolipoprotein E; MCI, mild cognitive impairment; SD, standard deviation; SOL-INCA, Study of Latinos-Investigation of Neurocognitive Aging.

**TABLE 2:** APOE alleles association with neurocognitivefunction in the SOL-INCA (additive inheritance mode)

Trait	*APOE* allele	OR [95% CI]	*P*-value
Significant cognitive decline	2	1.04 [0.85;1.29]	6.85E-01
	4	1.15 [1.01;1.32]	3.35E-02
MCI	2	0.78 [0.51;1.17]	2.30E-01
	4	0.94 [0.72;1.22]	6.19E-01

Notes: Models adjusted for sex, age, education, center, genetic background groups, and first five PCs. APOE *ε*3 used as the reference allele. Effect sizes and SEs were estimated based on the complex survey design method and were used toORsand 95% CIs. OR values larger than 1 represent decreasec neurocognitive function

Abbreviations: APOE, apolipoprotein E; CI, confidence interval; MCI, mild cognitive impairment; OR, odds ratio; PC, principal component; SOL-INCA, Study of Latinos-Investigation of Neurocognitive Aging; SE, standard error.

**TABLE 3:** APOE alleles association with neurocognitive function in the SOL-INCA by background groups (additive inheritance mode)

Trait	*APOE* allele	Cuban (n = 875)		Dominican (n = 424)		Mexican (n = 1411)		Puerto Rican (n = 734)		South American (n = 313)		Central American (n = 417)	
		OR [95% CI]	*P*-value	OR [95% CI]	*P*-value	OR [95% CI]	*P*-value	OR [95% CI]	*P*-value	OR [95% CI]	*P*-value	OR [95% CI]	*P*-value
Significant cognitive decline	2	1.20 [0.80; 1.80]	3.96E-01	0.98 [0.62;1.55]	9.46E-01	1.03 [0.69;1.54]	8.79E-01	1.06 [0.69; 1.62]	7.95E-01	1.93 [1.03;3.62]	7.17E-02	0.65 [0.30;1.38]	3.07E-01
	4	1.46 [1.13;1.88]	7.00E-03	0.95 [0.70;1.30]	7.69E-01	1.05 [0.77;1.43]	7.69E-01	1.04 [0.84;1.29]	7.31E-01	0.91 [0.55; 1.50]	7.39E-01	1.05 [0.76;1.46]	7.71E-01
MCI	2	0.98 [0.49;1.96]	9.54E-01	1.05 [0.40;2.72]	9.33E-01	0.54 [0.23;1.29]	2.19E-01	0.37 [0.16;0.84]	4.41E-02	0.74 [0.14;3.87]	7.85E-01	0.34 [0.05;2.19]	3.23E-01
	4	0.80 [0.42;1.53]	5.26E-01	0.98 [0.55;1.72]	9.43E-01	0.90 [0.58;1.38]	6.64E-01	1.04 [0.65;1.67]	8.64E-01	0.83 [0.39;1.76]	6.90E-01	1.08 [0.58;2.01]	8.34E-01

Notes: Models adjusted for sex, age, education, center, and first five PCs. Effect sizes and SEs were estimated based on the complex survey design method, and were used to compute ORs and 95% CIs. APOE *ε*3 used as the reference allele. OR values larger than 1 represent decreased neurocognitive function. *P*-values were estimated based on 10,000 permutations for each genetic background group. Heterogeneity *P*-values were not significant.

Abbreviations: APOE, apolipoprotein E; CI, confidence interval; MCI, mild cognitive impairment; OR, odds ratio; PC, principal component; SOL-INCA, Study of Latinos-Investigation of Neurocognitive Aging; SE, standard error.

**TABLE 4:** The interaction effect of proportion ancestry and APOE alleles on neurocognitive function in the SOL-INCA (additive inheritance mode)

Trait	*APOE* allele	Europe				Africa				Amerindian			
		Interaction		*APOE* allele		Interaction		*APOE* allele		Interaction		*APOE* allele	
		OR [95% CI]	*P*-value	OR [95% CI]	*P*-value	OR [95% CI]	*P*-value	OR [95% CI]	*P*-value	OR [95% CI]	*P*-value	OR [95% CI]	*P*-value
Significant cognitive decline	2	1.76 [0.70;4.40]	2.41E-01	0.77 [0.42;1.40]	4.06E-01	0.60 [0.22;1.61]	3.22E-01	1.23 [0.90;1.67]	2.03E-01	1.14 [0.32;4.05]	8.43E-01	1.08 [0.79;1.46]	6.48E-01
	4	1.58 [0.82;3.06]	1.90E-01	0.88 [0.58;1.33]	5.47E-01	1.20 [0.64;2.28]	5.77E-01	1.11 [0.93;1.34]	2.63E-01	0.47 [0.24;0.93]	3.83E-02	1.34 [1.11;1.63]	3.70E-03
MCI	2	0.47 [0.05;4.71]	5.50E-01	1.20 [0.30;4.83]	8.12E-01	4.63 [0.66;32.36]	1.57E-01	0.49 [0.24;1.01]	6.84E-02	0.03 [0.00;1.38]	1.07E-01	1.16 [0.60;2.25]	6.70E-01
	4	1.37 [0.38;4.92]	6.59E-01	0.81 [0.36; 1.85]	6.39E-01	0.76 [0.16;3.68]	7.50E-01	1.05 [0.72;1.52]	8.26E-01	1.01 [0.34;3.01]	9.92E-01	0.99 [0.66;1.50]	9.72E-01

Notes: Models adjusted for sex, age, education, center, and first five PCs. Effect sizes and SEs were estimated based on the complex survey design method and were used to compute ORs and 95% CIs. APOE *ε*3 used as the reference allele. Interaction *P*-values were estimated based on 10,000 permutations for each ancestry separately. OR values larger than 1 represent decreased neurocognitive function.

Abbreviations: APOE, apolipoprotein E; CI, confidence interval; MCI, mild cognitive impairment; OR, odds ratio; PC, principal component; SOL-INCA, Study of Latinos-Investigation of Neurocognitive Aging.

## RESEARCH IN CONTEXT

1. Systematic review: The authors reviewed the literature using traditional sources. Several publications have shown weak and inconsistent results for the association of the apolipoprotein E (*APOE*) alleles with cognitive decline, mild cognitive impairment (MCI), and Alzheimer’s disease-related dementias in Latinos, despite higher rates of these disorders compared to non-Latino Whites. Publications are appropriately cited.
2. Interpretation: Our findings suggest a differential association between the *APOE ε*2 and *APOE ε*4 alleles and risk for significant cognitive decline and MCI in the six Latino groups defined by country of origin. We also found that Amerindian genetic ancestry protects from the risk conferred by *APOE ε*4 on significant cognitive decline. Inconsistent estimated associations in Latinos may be due to different admixture patterns of continental ancestry across Latino groups.
3. Future directions: Future studies are needed to (a) identify enriched Amerindian genetic variants interacting with the *APOEε*4 allele, and the mechanism of interactions and (b) develop genetic measures for predicting significant cognitive decline and MCI in admixed individuals, potentially taking into account proportion ancestry or specific genetic variants.

## References

[1] Heron M Deaths: leading Causes for 2016. Natl Vital Stat Reports. 2016:67. 30248017

[2] Boyle PA, Wilson RS, Aggarwal NT, Tang Y, Bennett DA. Mild cognitive impairment: risk of Alzheimer disease and rate of cognitive decline. Neurology. 2006;67:441–445. 16894105 10.1212/01.wnl.0000228244.10416.20

[3] Wilson RS, Segawa E, Boyle PA, Anagnos SE, Hizel LP, Bennett DA. The natural history of cognitive decline in Alzheimer’s disease. Psychol Aging. 2012;27:1008–1017. 22946521 10.1037/a0029857 PMC3534850

[4] Cheng Y-W, Chen T-F, Chiu M-J. From mild cognitive impairment to subjective cognitive decline: conceptual and methodological evolution. Neuropsychiatr Dis Treat. 2017;13:491–498. 28243102 10.2147/NDT.S123428 PMC5317337

[5] Vega IE, Cabrera LY, Wygant CM, Velez-Ortiz D, Counts SE. Alzheimer’s Disease in the Latino community: intersection of genetics and social determinants of health. J Alzheimer’s Dis. 2017;58:979–992. 28527211 10.3233/JAD-161261 PMC5874398

[6] Alzheimer’s Association. 2018 Alzheimer’s disease facts and figures. Alzheimer’s Dement. 2018;14:367–429. 10.1016/j.jalz.2011.02.004 21414557

[7] Dilworth-Anderson P, Hendrie HC, Manly JJ, Khachaturian AS, Fazio S. Diagnosis and assessment of Alzheimer’s disease in diverse populations. Alzheimer’s Dement. 2008;4:305–309. 18631983 10.1016/j.jalz.2008.03.001

[8] Farrer LA, Cupples LA, Haines JL, Effects of age, sex, and ethnicity on the association between apolipoprotein E Genotype and Alzheimer disease. JAMA. 1997;278:1349–1356. 9343467

[9] Reas ET, Laughlin GA, Bergstrom J, Kritz-Silverstein D, Barrett-Connor E, McEvoy LK. Effects of APOE on cognitive aging in community-dwelling older adults. Neuropsychology. 2019;33:406–416. 30730162 10.1037/neu0000501 PMC6513314

[10] Makkar SR, Lipnicki DM, Crawford JD, APOE *ε*4 and the influence of sex, age, vascular risk factors, and ethnicity on cognitive decline. J Gerontol A Biol Sci Med Sci. 2020:1–11. 32396611 10.1093/gerona/glaa116 PMC7518559

[11] Ren D, Lopez OL, Lingler JH, Conley Y. The effect of the APOE *ε*2*ε*4 Genotype on the development of Alzheimer’s disease (AD) and Mild Cognitive Impairment (MCI) in Non-Latino Whites. J Am Geriatr Soc. 2020;68:1044–1049. 32017008 10.1111/jgs.16337 PMC7482099

[12] Liu C-C, Liu C-C, Kanekiyo T, Xu H, Bu G. Apolipoprotein E and Alzheimer disease: risk, mechanisms and therapy. Nat Rev Neurol. 2013;9:106–118. 23296339 10.1038/nrneurol.2012.263 PMC3726719

[13] Tang M-X, Stern Y, Marder K, The APOE-ε4 Allele and the Risk of Alzheimer Disease Among African Americans, Whites, and Hispanics. JAMA. 1998;279:751. 9508150 10.1001/jama.279.10.751

[14] Crean S, Ward A, Mercaldi CJ, Apolipoprotein E 4 prevalence in Alzheimer’s disease patients varies across global populations: a systematic literature review and meta-analysis. Dement Geriatr Cogn Disord. 2011;31:20–30. 21124030 10.1159/000321984

[15] Maestre G, Ottman R, Stern Y, Gurland B, Chun M, TangM-X, Apolipoprotein E and Alzheimer’s disease: ethnic variation in genotypic risks. Ann Neurol. 1995;37:254–259. 7847867 10.1002/ana.410370217

[16] Haan MN, Mungas DM, Gonzalez HM, Ortiz TA, Acharya A, Jagust WJ. Prevalence of dementia in older Latinos: the influence of type 2 diabetes mellitus, stroke and genetic factors. J Am Geriatr Soc. 2003;51:169–177. 12558712 10.1046/j.1532-5415.2003.51054.x

[17] O’Bryant SE, Johnson L, Reisch J, Risk factors for mild cognitive impairment among Mexican Americans. Alzheimers Dement. 2013;9:622–631.e1. 23643456 10.1016/j.jalz.2012.12.007 PMC3737282

[18] Campos M, Edland SD, Peavy GM. An exploratory study of APOE-*ε*4 Genotype and risk of Alzheimer’s disease in Mexican Hispanics NIH public access. J Am Geriatr Soc. 2013;61:1038–1040. 23772735 10.1111/jgs.12292 PMC3694500

[19] Romas SN, Santana V, Williamson J, Familial Alzheimer disease among Caribbean Hispanics. Arch Neurol. 2002;59:87–91. 11790235 10.1001/archneur.59.1.87

[20] González HM, Tarraf W, Jian X, Apolipoprotein E genotypes among diverse middle-aged and older Latinos: study of Latinos-investigation of neurocognitive aging results (HCHS/SOL). Sci Rep. 2018;8. 30546063 10.1038/s41598-018-35573-3 PMC6292877

[21] Lavange LM, Kalsbeek WD, Sorlie PD, Sample design and cohort selection in the Hispanic community health study/study of Latinos. Ann Epidemiol. 2010;20:642–649. 20609344 10.1016/j.annepidem.2010.05.006 PMC2921622

[22] Sorlie PD, Avilé S-Santa LM, Wassertheil-Smoller S, Design and implementation of the Hispanic community health study/study of Latinos. Ann Epidemiol. 2010;20:629–641. 20609343 10.1016/j.annepidem.2010.03.015 PMC2904957

[23] Conomos MP, Laurie CA, Stilp AM, Genetic diversity and association studies in US Hispanic/Latino populations: applications in the Hispanic community health study/study of Latinos. Am J Hum Genet. 2016;98:165–184. 26748518 10.1016/j.ajhg.2015.12.001 PMC4716704

[24] González HM, Tarraf W, Schneiderman N, Prevalence and correlates of mild cognitive impairment among diverse Hispanics/Latinos: study of Latinos-investigation of neurocognitive aging results. Alzheimer’s Dement. 2019;15:1507–1515. 31753701 10.1016/j.jalz.2019.08.202 PMC7318558

[25] Callahan Christopher M, Unverzagt Frederick W, Hui Siu L, Perkins Anthony J, Hendrie Hugh C. Six-item screener to identify cognitive impairment among potential subjects for clinical research. Med Care. 2002;40(9):771–781. 10.1097/00005650-200209000-00007. 12218768

[26] González HM, Mungas D, Reed BR, Marshall S, Haan MN. A new verbal learning and memory test for English- and Spanish-speaking older people. J Int Neuropsychol Soc. 2001;7:544–555. 11459106 10.1017/s1355617701755026

[27] Lezak MD, Howieson DB, Loring DW, Hannay HJ, Fischer JS. Neuropsychological Assessment, PsycNET. 4th ed. New York: Oxford University Press; 2004.

[28] Tomaszewski Farias S, Mungas D, Harvey DJ, Simmons A, Reed BR, Decarli C. The measurement of everyday cognition: development and validation of a short form of the everyday cognition scales. Alzheimers Dement. 2011;7:593–601. 22055976 10.1016/j.jalz.2011.02.007 PMC3211103

[29] González HM, Tarraf W, Gouskova N, Neurocognitive function among middle-aged and older Hispanic/Latinos: results from the Hispanic community health study/study of Latinos. Arch Clin Neuropsychol. 2015;30:68–77. 25451561 10.1093/arclin/acu066 PMC4351363

[30] González HM, Tarraf W, Fornage M, A research framework for cognitive aging and Alzheimer’s disease among diverse US Latinos: design and implementation of the Hispanic community health study/study of Latinos—investigation of neurocognitive aging (SOL-INCA). Alzheimer’s Dement. 2019;15:1624–1632. 31759880 10.1016/j.jalz.2019.08.192 PMC6925624

[31] Albert MS, DeKosky ST, Dickson D, The diagnosis of mild cognitive impairment due to Alzheimer’s disease: recommendations from the National Institute on Aging-Alzheimer’s Association workgroups on diagnostic guidelines for Alzheimer’s disease. Alzheimers Dement. 2011;7:270–279. 21514249 10.1016/j.jalz.2011.03.008 PMC3312027

[32] Sofer T, Wong Q, Hartwig FP, Genome-wide association study of blood pressure traits by Hispanic/Latino background: the Hispanic community health study/study of Latinos. Sci Rep. 2017;7: 10348. 28871152 10.1038/s41598-017-09019-1 PMC5583292

[33] Conomos MP, Reiner AP, Weir BS, Thornton TA. Model-free estimation of recent genetic relatedness. Am J Hum Genet. 2016;98:127–148. 26748516 10.1016/j.ajhg.2015.11.022 PMC4716688

[34] Alexander DH, Novembre J, Lange K. Fast model-based estimation of ancestry in unrelated individuals. Genome Res. 2009;19:1655–1664. 19648217 10.1101/gr.094052.109 PMC2752134

[35] Lumley T, Scott A. Tests for regression models fitted to survey data. Aust New Zeal J Stat. 2014;56:1–14.

[36] Nelson SC, Doheny KF, Pugh EW, Romm JM, Ling H, Laurie CA, Imputation-based genomic coverage assessments of current human genotyping arrays. G3 (Bethesda). 2013;3:1795–1807. 23979933 10.1534/g3.113.007161 PMC3789804

[37] Karr JE, Graham RB, Hofer SM, Muniz-Terrera G. When does cognitive decline begin? A systematic review of change point studies on accelerated decline in cognitive and neurological outcomes preceding mild cognitive impairment, dementia, and death. Psychol Aging. 2018;33:95–218. 10.1037/pag0000236 PMC5906105 29658744

[38] Rodríguez JJL, Cepero AV, Gil IYS, Incidence of dementia and association with APOE genotype in older Cubans. Dement Neuropsychol. 2014;8:356–363. 29213926 10.1590/S1980-57642014DN84000009 PMC5619184

[39] Cornejo-Olivas M, Rajabli F, Marca V, Dissecting the role of Amerindian genetic ancestry and ApoE *ε*4 allele on Alzheimer disease in an admixed Peruvian population. BioRxiv. 2020. 10.1016/j.neurobiolaging.2020.10.003 PMC8122013 33541779

[40] Rajabli F, Feliciano BE, Celis K, Ancestral origin of ApoE *ε*4 Alzheimer disease risk in Puerto Rican and African American populations. PLoS Genet. 2018;14. 10.1371/journal.pgen.1007791 PMC6281216 30517106

[41] Blue EE, Horimoto ARVR, Mukherjee S, Wijsman EM, Thornton TA. Local ancestry at APOE modifies Alzheimer ‘ s disease risk in Caribbean Hispanics. Alzheimer’s Dement. 2019:1–9. 31606368 10.1016/j.jalz.2019.07.016 PMC6925639

[42] Wu L, Zhao L. ApoE2 and Alzheimer’s disease: time to take a closer look. Neural Regen Res. 2016;11:412–413. 27127474 10.4103/1673-5374.179044 PMC4829000

[43] Jiang Y, He T, Deng W, Sun P. Association between apolipoprotein E gene polymorphism and mild cognitive impairment: a meta-analysis. Clin Interv Aging. 2017;12:1941–1949. 29180857 10.2147/CIA.S143632 PMC5691922

[44] Caselli RJ, Reiman EM, Locke DEC, Cognitive domain decline in healthy apolipoprotein E *ε*4 Homozygotes before the diagnosis of mild cognitive impairment. Arch Neurol. 2007;64:1306. 17846270 10.1001/archneur.64.9.1306

[45] Eid A, Mhatre I, Richardson JR. Gene-environment interactions in Alzheimer’s disease: a potential path to precision medicine. Pharmacol Ther. 2019;199:173–187. 30877021 10.1016/j.pharmthera.2019.03.005 PMC6827882

