# Supplementary material for: APOE alleles’ association with cognitive function differs across Hispanic/Latino groups and genetic ancestry in the study of Latinos-investigation of neurocognitive aging (HCHS/SOL)
Source: Alzheimers Dement. Author manuscript; Available in PMC 2021 Apr 2. (PMC8016734; doi:10.1002/alz.12205)
Supplement: Appendix_1 [file NIHMS1677351-supplement-Appendix_1.docx]

**Appendix 1:**

We analyzed an additional cognitive outcome, a continuous indicator of cognitive decline previously described ^1^, which was used to create the significant cognitive decline outcome analyzed in the primary analysis. This cognitive outcome ranges from -4.69 to 5.83 with a normal distribution around the mean (0.045).

**Results**

Table 1 summarizes the associations between the *APOE* alleles and continuous cognitive decline outcome. The continuous decline outcome shows a significant decrease (meaning a larger cognitive decline) in individuals with one or more copies of *APOE*-ε4, compared to individuals without *APOE*-ε4 allele (Beta=-0.11, p-value=0.009).

**Table 1:** *APOE* alleles association with continuous cognitive decline in the SOL-INCA (additive inheritance mode). Models adjusted for sex, age, education, center, genetic background groups, and first five PCs. *APOE*-ε3 used as the reference allele. Effect sizes and SEs were estimated based on the complex survey design method and were used to compute 95% confidence intervals (CIs). Negative values of beta represent a larger cognitive decline.

Further stratification to the six Latino background groups shows the association remains significant only for Cubans showing a stronger effect on the continuous cognitive decline compared to the effect in the total population (Beta= -0.25, p-value= 0.015). In the Dominican group the *APOE*-ε2 allele protects from continuous cognitive decline (Beta= 0.37, p-value= 0.01). Heterogeneity tests for differences between the background groups’ association tests were significant for both *APOE*-ε2 (p-value <0.0008) and for *APOE*-ε4 (p-value <0.05).

**Table 2:** *APOE* alleles association with continuous cognitive decline in the SOL-INCA by background groups (additive inheritance mode). Models adjusted for sex, age, education, center, and first five PCs. Effect sizes and SEs were estimated based on the complex survey design method and were used to compute 95% confidence intervals (CIs). *APOE*-ε3 used as the reference allele. Negative values of beta represent a larger cognitive decline. P-values were estimated based on 10,000 permutations for each genetic background group.

Proportion ancestry interaction with *APOE* alleles' effects on neurocognitive traits is presented in Table 5. A significant interaction effect was found between African ancestry with *APOE*-ε2 on continuous cognitive decline (Beta=0.65, p-value= 0.04), presenting higher protection from continuous cognitive decline in *APOE*-ε2 carriers associated with higher African proportion. Also, a significant interaction effect was found between Amerindian ancestry with *APOE*-ε4 on continuous cognitive decline ( Beta= 0.45, p-value= 0.04), presenting a lower risk for cognitive decline in *APOE*-ε4 carriers associated with higher Amerindian proportion.

**Table 3:** The interaction effect of proportion ancestry and *APOE* alleles on continuous cognitive decline in the SOL-INCA (additive inheritance mode). Models adjusted for sex, age, education, center, and first five PCs. Effect sizes and SEs were estimated based on the complex survey design method and were used to compute 95% confidence intervals (CIs). *APOE*-ε3 used as the reference allele. Interaction P-values were estimated based on 10,000 permutations for each ancestry separately. Negative values of beta represent a larger cognitive decline.

**Discussion:**

Overall, the association results of *APOE* alleles with continuous cognitive decline are similar to the results for the significant cognitive decline trait. The main result shows an association between the *APOE*-ε4 allele and the risk of continuous cognitive decline in the total population. In the stratified analysis, this result remained significant only for Cubans. An additional protective association was found between the *APOE*-ε2 allele and continuous cognitive decline in the Dominican group only. Significant heterogeneity tests suggest differential effects of both *APOE* alleles on the continuous cognitive decline across the Latino background groups.

Increased proportion of genetic Amerindian ancestry was associated with a protective effect from the risk of *APOE*-ε4 on continuous cognitive decline, compatible with the significant cognitive decline results. The analysis also revealed an interaction effect presenting higher protection from cognitive decline in *APOE*-ε2 carriers associated with a higher African proportion. We infer that ancestry-specific genetic variants may explain the differential effects of *APOE* alleles in the six Latino backgrounds.

**References:**

1. González, H. M. *et al.* A research framework for cognitive aging and Alzheimer’s disease among diverse US Latinos: Design and implementation of the Hispanic Community Health Study/Study of Latinos—Investigation of Neurocognitive Aging (SOL-INCA). *Alzheimer’s Dement.* **15**, 1624–1632 (2019).
