# Supplementary figures and images for: APOE alleles’ association with cognitive function differs across Hispanic/Latino groups and genetic ancestry in the study of Latinos-investigation of neurocognitive aging (HCHS/SOL)
Source: Alzheimers Dement. Author manuscript; Available in PMC 2021 Apr 2. (PMC8016734; doi:10.1002/alz.12205)

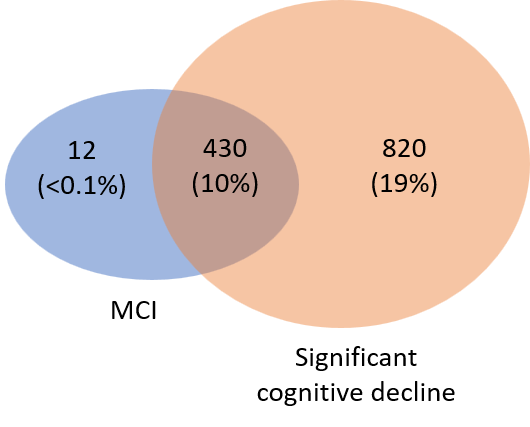

Supplement: Supplementary_Figure_1 [file NIHMS1677351-supplement-Supplementary_Figure_1.tif]
